# Effects of transcutaneous tibial nerve stimulation on females with overactive bladder syndrome in multiple sclerosis a protocol for a systematic review and meta-analysis

**DOI:** 10.1371/journal.pone.0269371

**Published:** 2022-07-28

**Authors:** Huan Tu, Ning Li, Wanna Liu, Zhonghe Fan, Dezhi Kong

**Affiliations:** 1 School of Sports Medicine and Health, Chengdu Sport University, Chengdu, Sichuan, PR China; 2 Institute of Sports Medicine and Health, Chengdu Sport University, Chengdu, Sichuan, PR China; Mugla Sitki Kocman Universitesi, TURKEY

## Abstract

**Background:**

Overactive bladder (OAB) is a problem that increasingly affects adults and the elderly, especially women. It may affect quality of life, ability to participate and overall wellbeing. Transcutaneous tibial nerve stimulation (TTNS) is a form of neuromodulation involving the use of electrical impulses to address urinary symptoms. There are many randomized controlled trials that have shown that TTNS is effective at treating overactive bladder. In recent years, TTNS has gained increasing attention for this condition. But its effect in females remains controversial and evidence is lacking. Therefore, the main purpose of this study will be to systematically evaluate the effect of TTNS on females with OAB in Multiple sclerosis (MS) by conducting a systematic review and meta-analysis, and also to provide a reference for the application of TTNS in OAB.

**Methods:**

A systematic review of eligible articles will be conducted using Preferred Reporting Items for Systematic Reviews and Meta-analysis (PRISMA) guidelines. A comprehensive search of the literature will be conducted in PubMed, Web of Science, The Cochrane Library, Chinese National Knowledge Infrastructure Database (CNKI), Wanfang Data, Weipu Electronics, and other databases. We will include randomized controlled trials about TTNS in females with OAB in MS. Two reviewers will screen titles, abstracts, and full texts independently. We will use a hierarchy of recommended assessment, development, and assessment methods to assess the overall certainty of the evidence and report findings accordingly. Endnote X9 will be used to select the studies and Review Manager V.5.4 (Cochrane Collaboration) will be used to conduct the meta-analysis. The mean difference or standard deviation with 95% confidence interval (CI) will be used in the computation of continuous variables to synthesize data.

**Results:**

The results will provide evidence for judging whether TTNS is effective in females with OAB and MS.

**Conclusion:**

This study will provide reliable evidence for the effect of TTNS in female patients with OAB and MS.

**Trial registration:**

**Systematic review registration:** PROSPERO **CRD42021256861**.

## Introduction

Multiple sclerosis (MS) is a chronic inflammatory disease which results in many functional disorders [1, 2]. And lower urinary system dysfunction (LUSD) is a common problem in female patients with MS [3], about 7–10% of patients report lower urinary system dysfunction as an initial symptom, and 60–80% report it during the course of the disease [4].

Overactive bladder (OAB) is a clinical syndrome characterized by acute urinary storage symptoms, with or without acute urinary incontinence, but often accompanied by frequent urination and nocturia, is the most frequent lower urinary system dysfunction in the total daytime conditions, which affects a large proportion of women [4–6]. OAB is common in patients with multiple sclerosis with a limited number of treatment options. Therapeutic agents consist primarily of antimuscarinic and β-agonist medications, as well as off-label use of antidepressants in some cases. Although these drugs are effective in improving the symptoms, they are likely to have adverse side effects, such as facial flushing, constipation, and hyperthermia [7].

In recent years, many studies have found that electrical stimulation can be used as third-line therapy and this method can be applied noninvasively or with a surgical implant, such as transcutaneous sacral electrical stimulation 、transcranial direct current stimulation and repetitive transcranial magnetic stimulation [6, 8–11]. Transcutaneous tibial nerve stimulation is known to be an effective way to improve OAB symptoms, and can be used clinically as it is low cost and non-invasive with minimal side effects [12]. And some studies have shown that transcutaneous tibial nerve stimulation therapy has emerged as a new alternative in the management of patients with OAB [12], which is known to be an effective way to improve OAB symptoms, and can be used clinically as it is low cost and non-invasive with minimal side effects. These studies concluded that TTNS is a safe and effective treatment of women with OAB by conducting randomized controlled trials [3, 12, 13], however, there is no evidence-based basis for this topic.

Therefore, the main purpose of this study is to evaluate the effect of TTNS on female patients with OAB in MS. In addition, suggestions will be made for future research in this field based on the results of this study.

## Materials and methods

### Study registration

The study protocol has been registered with the International Prospective Register of Systematic Reviews (PROSPERO) (registration number: CRD42021256861). The protocol is reported strictly according to the Preferred Reporting Items for Meta-Analyses Protocols (PRISMA-P) guidelines [14].

### Eligibility criteria

#### Type of study

We will include randomized controlled trials of TTNS in females with OAB and MS.

#### Type of participant

Female MS patients with symptoms related to overactive bladder who are older than the age of 18 (following the clinical diagnostic criteria for OAB and multiple sclerosis) Overactive bladder was defined according to the ICS definition as “urinary urgency, usually accompanied by frequency and nocturia, with or without urgency urinary incontinence, in the absence of urinary tract infection or other obvious pathology” [15, 16] There will be no restrictions on participants’ race or nationality.

#### Type of intervention

Our research will include studies in which the experimental group received conventional rehabilitation training and transcutaneous tibial nerve stimulation, while the control group received only conventional rehabilitation therapy. In these studies, TTNS was performed using a stimulator. Two carbon rubber electrodes were placed below the left medial malleolus and 5 cm proximal to the distal electrode. The big toe was in plantar-flexion during stimulation and the stimulus intensity was according to the patient’s tolerance. After placing the electrode, the stimulation amplitude was reduced to a level just below the somatic sensory threshold.

#### Types of outcome measurements

The primary outcome will be used to assess the improvement of OAB, and the main evaluation index will be a voiding diary(including symptoms of urgency, frequency, nocturia, amount of leakage)、Overactive Bladder Symptom Score (OABSS) 、Overactive Bladder-Validated 8- question Awareness Tool (OAB-V8)、Overactive bladder questionnaire (OBQ). The secondary outcome will be used to assess the quality of life which including Health Related Quality of Life (HRQoL). And others relevant indicators will be included for reference. Any study using a tool other than those mentioned above to evaluate improvement of OAB and QOL, such as other QOL questionnaires and OAB questionnaires, will be excluded from analysis.

#### Inclusion criteria

We will use the PICOS (Population, Intervention, Comparison, Outcome, and Study design) model to select studies for this review. The inclusion criteria are as follows:

1. Participants: female patients with Overactive Bladder Syndrome and Multiple sclerosis;
2. Intervention: the experimental group received conventional rehabilitation training and TTNS;
3. Comparator: the control group received conventional rehabilitation therapy;
4. Outcomes: the primary outcome is the improvement of OAB, and the secondary outcome is the quality of life;
5. Study design: Randomized clinical trial.

### Exclusion criteria

1. Outcome index data are missing;
2. Repeated publication, too little information, incomplete data and abstract only; no full text.

### Search methods for identification of studies

#### Electronic data sources

The following electronic databases will be searched from inception to now: PubMed, The Cochrane Library, Web of Science, China National Knowledge Infrastructure (CNKI), WanFang Data, and Weipu Electronics. The reference lists of the included studies will be manually searched to identify additional relevant studies.

#### Other resources

Relevant references will be reviewed and screened. In addition, we will email to authors if the article is not complete or data are missing.

#### Search strategy

Two reviewers will independently extract data and perform quality assessment of the included studies. Studies concerning the effects of TTNS on females with OAB in MS will be included in this systematic review and meta-analysis. Only randomized controlled trials will be included in the meta-analysis. Detailed information about the PubMed search strategy is presented in Table 1.

**Table 1:** Search strategy for the PubMed database.

Number	Search items
1	Multiple sclerosis
2	MS
3	Disseminated Sclerosis
4	Focal sclerosis
5	Insular sclerosis
6	Multilocular sclerosis
7	Multiple sclerosis or MS or Disseminated Sclerosis or Focal sclerosis or Insular sclerosis or Multilocular sclerosis
8	Overactive bladder symptoms
9	Overactive bladder
10	OAB
11	OA
12	Overactive bladder symptoms or Overactive bladder or OAB or OA
13	Transcutaneous tibial nerve stimulation
14	TTNS
15	transcutaneous posterior tibial nerve stimulation
16	TPTNS
17	Percutaneous tibial nerve stimulation
18	PTNS
19	Transcutaneous tibial nerve stimulation or TTNS or transcutaneous posterior tibial nerve stimulation or TPTNS or Percutaneous tibial nerve stimulation or PTNS
20	Randomized controlled trial
21	Randomized
22	Clinical trial
23	Randomly
24	Controlled clinical trials
25	Controlled before-after studies
26	Randomized controlled trial or Randomized or Clinical trial or Randomly or Controlled clinical trials or Controlled before-after studies
27	Multiple sclerosis or MS or Disseminated Sclerosis or Focal sclerosis or Insular sclerosis or Multilocular sclerosis and Overactive bladder symptoms or Overactive bladder or OAB or OA and Transcutaneous tibial nerve stimulation or TTNS or transcutaneous posterior tibial nerve stimulation or TPTNS or Percutaneous tibial nerve stimulation or PTNS and Randomized controlled trial or Randomized or Clinical trial or Randomly or Controlled clinical trials or Controlled before-after studies

### Data collection

#### Selection of studies

**Fig 1:**
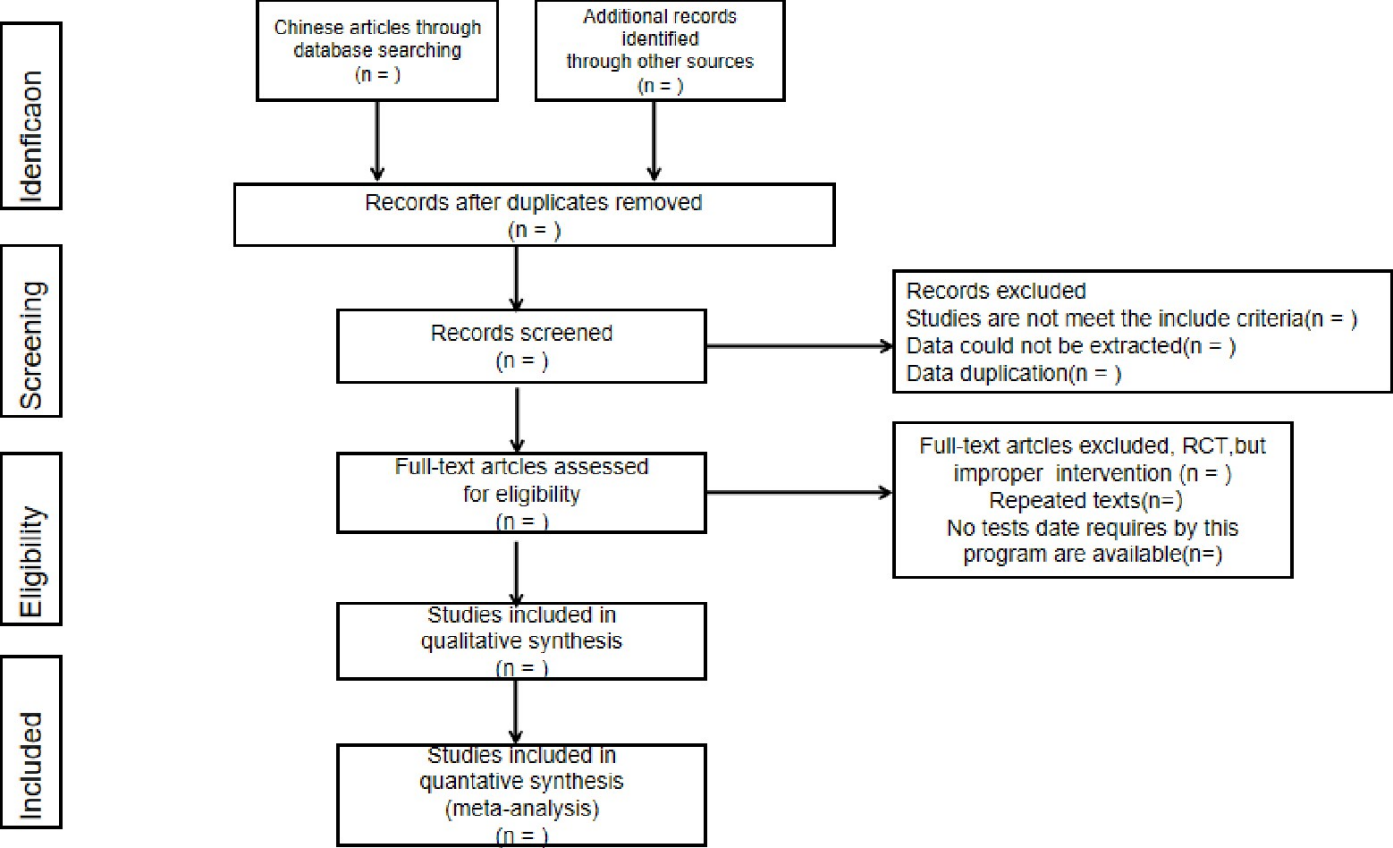
Flow chart of the study. Adapted from Preferred Reporting Items for Systematic Reviews and Meta-Analyses Protocols (PRISMA-P). RCT = Randomized controlled trial.

The retrieved studies will be imported into Endnote software 9.1 to remove duplicates. Two researchers (LN and KDZ) will screen the titles and abstracts independently according to the pre-established inclusion and exclusion criteria. After that, the full text will be screened. Two researchers will crosscheck the included studies, and a third researcher (TH) will be involved if disagreements occur. The study selection process is demonstrated in a PRISMA flow diagram (Fig 1 Flow chart and descriptions of study selection).

#### Data extraction and management

The other two researchers (TH and LWN) will extract data independently to fill out the predesigned form. The information will include author, country, publication year, methodological quality, characteristics of participants, details about the intervention and comparisons, outcomes, the specific data, results, conclusions, follow-up, adverse events, conflicts of interest, sources of funds, and ethical approval. The extracted data will be crosschecked by the two researchers. A third researcher (LN) will be involved if any disagreement arises. The authors of the included studies will be contacted for further information when necessary.

#### Risk assessment of bias included in the study

The risk of bias will be evaluated using a randomized controlled trial bias risk assessment tool recommended by the International Cochrane Manual 5.1.0. The main components of the literature quality assessment will include: (1) whether group allocation was random; (2) whether concealment was used; (3) blindness (participants, implementers, evaluators); (4) integrity of the resulting data; (5) selective reporting; (6) other sources of bias. This will be evaluated independently by two researchers (TH and WNL). We will rate the risk of bias according to three levels: when none of the criteria are met, it will be regarded as high; when all criteria are met, it will be regarded as low; when a study presents insufficient information to determine the risk, it will be regarded as unclear. After assessment, the results will be crosschecked by two researchers (DZK and ZHF). The third researcher (LN) will be involved if a disagreement occurs.

### Data synthesis

Review Manager software (RevMan5.4) will be used to conduct all data analyses if it is possible to perform a meta-analysis. Effect calculation: a study using the same results, the mean difference (MD), and the corresponding 95% confidence interval (CI). The *x*^2^ test will be used to test whether the combined statistics of multiple similar studies are significant, and the P-value of the statistic will be worth according to the *x*^2^. If *P*<0.05, then the combined statistics of multiple studies will be considered significant; if *P*>0.05, the combined statistics of multiple studies will be considered not significant. Heterogeneity test: heterogeneity of the intervention effect is inevitable, because of the differences in the design of the studies. The heterogeneity among the results will be analyzed by *x*^2^ test (*P* = 0.10), combined with *I^2^* to make a quantitative judgment of heterogeneity. If *P*>0.10 and *I^2^*<50%, this will indicate that the studies are homogeneous, and a fixed effect model should be selected; if *P*≤0.10 and *I^2^*>50%, this will indicate heterogeneity among the studies, and a random effect model should be selected. The level of significance in the meta-analysis will be set at *P*<0.05. In the case of obvious heterogeneity, subgroup analysis or sensitivity analysis will be used, or descriptive analysis.

#### Management of missing data

The relevant corresponding author will be contacted if there are insufficient or missing data. If accurate data is still unavailable after contacting the corresponding author, the study will be excluded.

#### Assessment of reporting biases

If more than 10 RCTs are included, funnel plots will be used to evaluate the potential publication bias.

#### Subgroup analysis

If there is large heterogeneity in the included studies, subgroup analyses will be performed on different conventional rehabilitation programs with TTNS, such as pelvic floor muscle training. If necessary, the time of therapy and the stimulus intensity will be analyzed.

#### Sensitivity analysis

Sensitivity analysis will be conducted by excluding studies one by one, so that we can determine the source of heterogeneity.

#### Grading the quality of evidence

This paper will use the evidence quality rating method to evaluate the results obtained from this analysis. GRADE will be assessed across the domains of risk of bias, consistency, directness, precision, and publication bias. In the context of the system review, quality reflects our confidence in the effectiveness of the assessment. It has four evaluation levels, namely, high (further research is very unlikely to change our confidence in the estimate of effect), moderate (further research is likely to have an important impact on our confidence in the estimate of effect and may change the estimate), low (further research is very likely to have an important impact on our confidence in the estimate of effect and change the estimate), or very low (very uncertain about the estimate of effect).

#### Ethics and dissemination

Ethical approval is not required due to the nature of this meta-analysis, which is based on published papers. The results of this systematic review and meta-analysis will be published in a peer-reviewed journal once we finish the study.

## Discussion

Patients who have multiple sclerosis are highly likely to suffer from lower urinary tract symptoms [17, 18]. And many patients have overactive bladder syndrome, which has a major pejorative effect on quality of life [19, 20]. In recent years, TTNS has been widely applied to patients with overactive bladder syndrome and multiple sclerosis [21–24]. But there have been no systematic reviews or meta-analyses on the effect of TTNS on females with overactive bladder syndrome in multiple sclerosis.

This study will assess the prognostic impact of TTNS in females with overactive bladder syndrome and multiple sclerosis, via literature collection, literature screening, data extraction and data analysis. This study may provide some guidance for other researchers studying the treatment effects of TTNS in females with overactive bladder syndrome in multiple sclerosis in the future.

However, the absence of sufficient randomized controlled trials may be a limitation for this meta-analysis, and the inclusion criteria do not take into account the different courses of disease and age, which will affect the meta-analysis; in addition, the treatment frequency of TTNS may be another limitation of the study. During the analysis, we will try to avoid these problems through careful literature screening and subgroup analysis.

## Supporting information

S1 Checklist PRISMA-P (Preferred Reporting Items for Systematic review and Meta-Analysis Protocols) 2015 checklist: Recommended items to address in a systematic review protocol*. (DOC)

S1 File Proof of funding support. (DOC)
