# Supplementary material for: Effects of transcutaneous tibial nerve stimulation on females with overactive bladder syndrome in multiple sclerosis a protocol for a systematic review and meta-analysis
Source: PLoS One. 2022 Jul 28;17(7):e0269371. doi: 10.1371/journal.pone.0269371 (PMC9333199; doi:10.1371/journal.pone.0269371)
Supplement: S1 File — (DOC) [file pone.0269371.s002.doc]

**Proof of Funding Support**

《PLOS ONE》editorial office：

This is to have our institution *Huang Tu, Ning Li, Wanna Liu*, and other authors write the title as Effects of transcutaneous tibial nerve stimulation on female with Overactive Bladder Syndrome in Multiple Sclerosis A protocol for a systematic review and meta-analysis. After the review of our institution, there is no confidential content involved, and the author's signature and the order can be published publicly without dispute. We agree to recommend the situation that the article published by your journal has not been submitted for more than one draft. This work is supported by the Key Laboratory of Sports Medicine of Sichuan Province, Institute of Sports Medicine and Health, Chengdu Sport University (Approval No.: 2021-A013).

Hereby to certify

Institute of Sports Medicine and Health,

Chengdu Sport University

May 6, 2022
